# A ‘Bit’ of Appendicitis: A Case of a Foreign Object in the Adult Appendix

**DOI:** 10.7759/cureus.4751

**Published:** 2019-05-24

**Authors:** Elizabeth Packard, Andrew Groff, Zainab Shahid, Nitasa Sahu, Rohit Jain

**Affiliations:** 1 Internal Medicine, Penn State Health Milton S. Hershey Medical Center, Hershey, USA; 2 Internal Medicine, Lake Erie College of Osteopathic Medicine, Erie, USA

**Keywords:** foreign body, appendicitis, perforation, appendectomy, drill bit

## Abstract

Appendicitis is the most common abdominal surgical emergency, and if left untreated, can lead to an abscess, peritonitis, or even death. The exact mechanism of appendicitis has yet to be elucidated, but the predominant theory is that different forms of luminal obstruction of the vermiform appendix lead to ischemia of the appendix wall and subsequent translocation of bacteria across the compromised mucosa, leading to transmural inflammation. The most common etiology is hyperplasia of lymphoid tissue in the mucosa, often secondary to infection and inflammation with gradual symptom onset. Rarer causes of obstruction include parasitic infiltration, fibrous bands, carcinoid syndrome, and foreign body ingestion and often have atypical or absent symptomatology, making diagnosis more challenging and complications more frequent. We present a rare case of foreign body-associated appendicitis with distal lodging in the appendix and highlight the importance of prophylactic appendectomy to avoid severe complications.

## Introduction

Acute appendicitis is inflammation of the vermiform appendix and classically presents with periumbilical pain that progresses to the right lower quadrant, fever, nausea, vomiting and rebound tenderness. Appendicitis is most commonly caused by a bacterial infection or inflammation, and patients often present with this classic symptomatology. The lifetime risk of appendicitis is seven to nine percent, with complications including abscess, peritonitis and even death occurring in 16-24% of cases [1-5]. Therefore, early diagnosis and treatment is critical to prevent morbidity and mortality.

Appendicitis caused by foreign body ingestion is a rare occurrence in adults. Most ingested foreign bodies pass through the GI tract without incident, with less than one percent impacting in the appendix [3]. Within the GI tract, the dependent position of the cecum and its slower motility make it susceptible to the settlement of foreign bodies. The chance of entry into the appendix is dependent upon the position of the appendix and the size of the appendiceal orifice. Once in the appendix, foreign bodies are often unable to be expelled back into the cecum due to insufficient peristaltic motility [6,7]. Some ingested foreign bodies remain dormant within the appendix without inciting inflammation for up to several years [4]. Blunt objects usually remain dormant for longer periods of time and cause appendicitis due to progressive obstruction. In contrast, ingestion of sharp objects is associated with higher rates of complication including perforation and peritonitis.

## Case presentation

**Figure 1:**
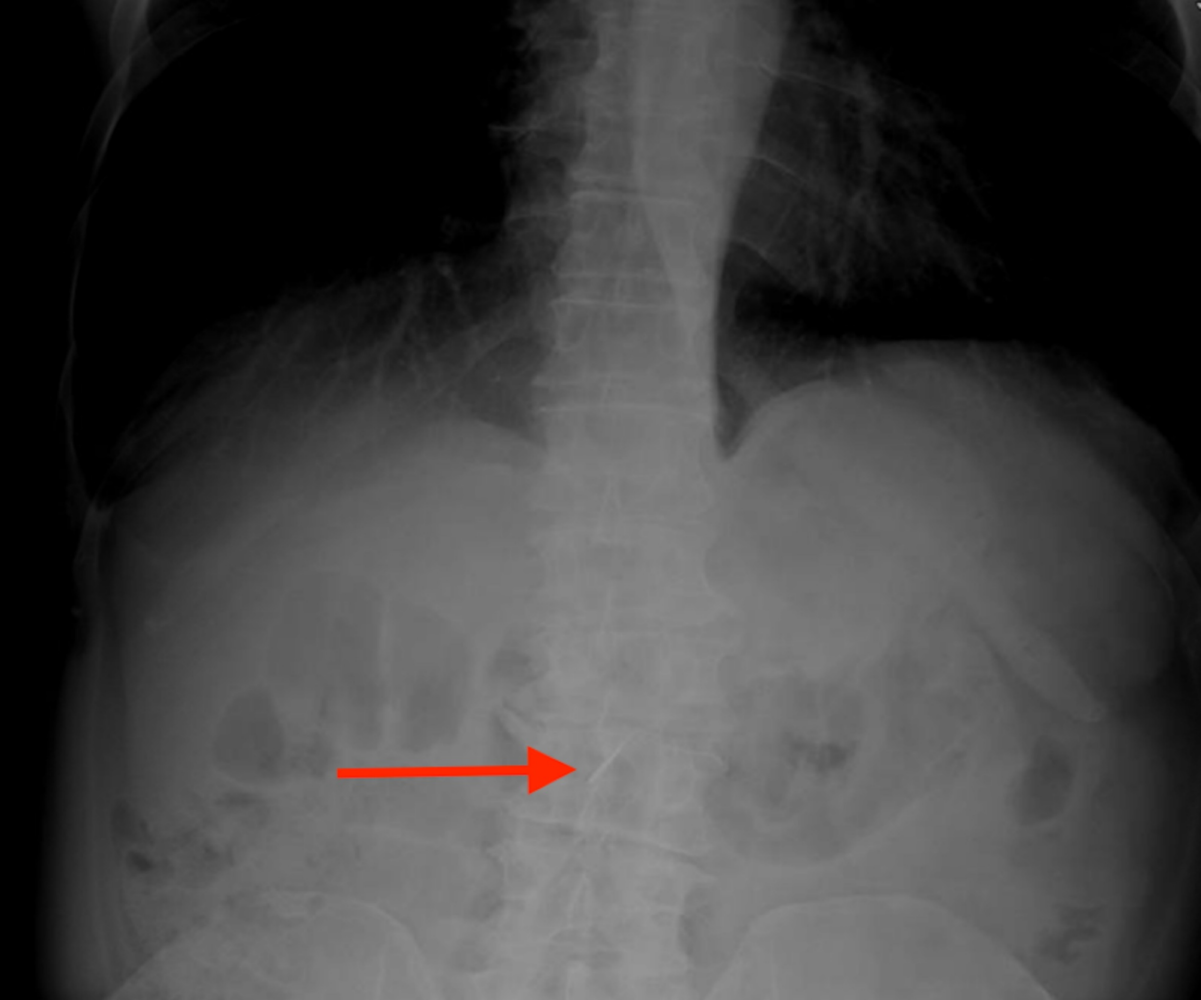
Abdominal X-ray revealing a thin, 2.3 cm radiopaque foreign body (red arrow) overlying the mid abdomen, likely representative of drill bit.

A 60-year-old male with no significant past medical history presented to the emergency department for evaluation after swallowing a foreign body while at the dentist. The patient reported that he was at the dentist getting a tooth filling when the drill bit accidentally fell out. He described the drill bit as approximately one inch long and with a sharp end. He endorsed a globus sensation in his throat; however, he denied associated pain. The patient was afebrile, had a pulse of 72 beats per minute, a blood pressure of 135/102 mmHg, a respiratory rate of 18 breaths per minute, and an oxygen saturation of 99% on room air. Laboratory workup was unremarkable, including comprehensive metabolic panel and complete blood count. Abdominal X-ray revealed a thin radiopaque foreign body overlying the mid abdomen (Figure 1).

**Figure 2:**
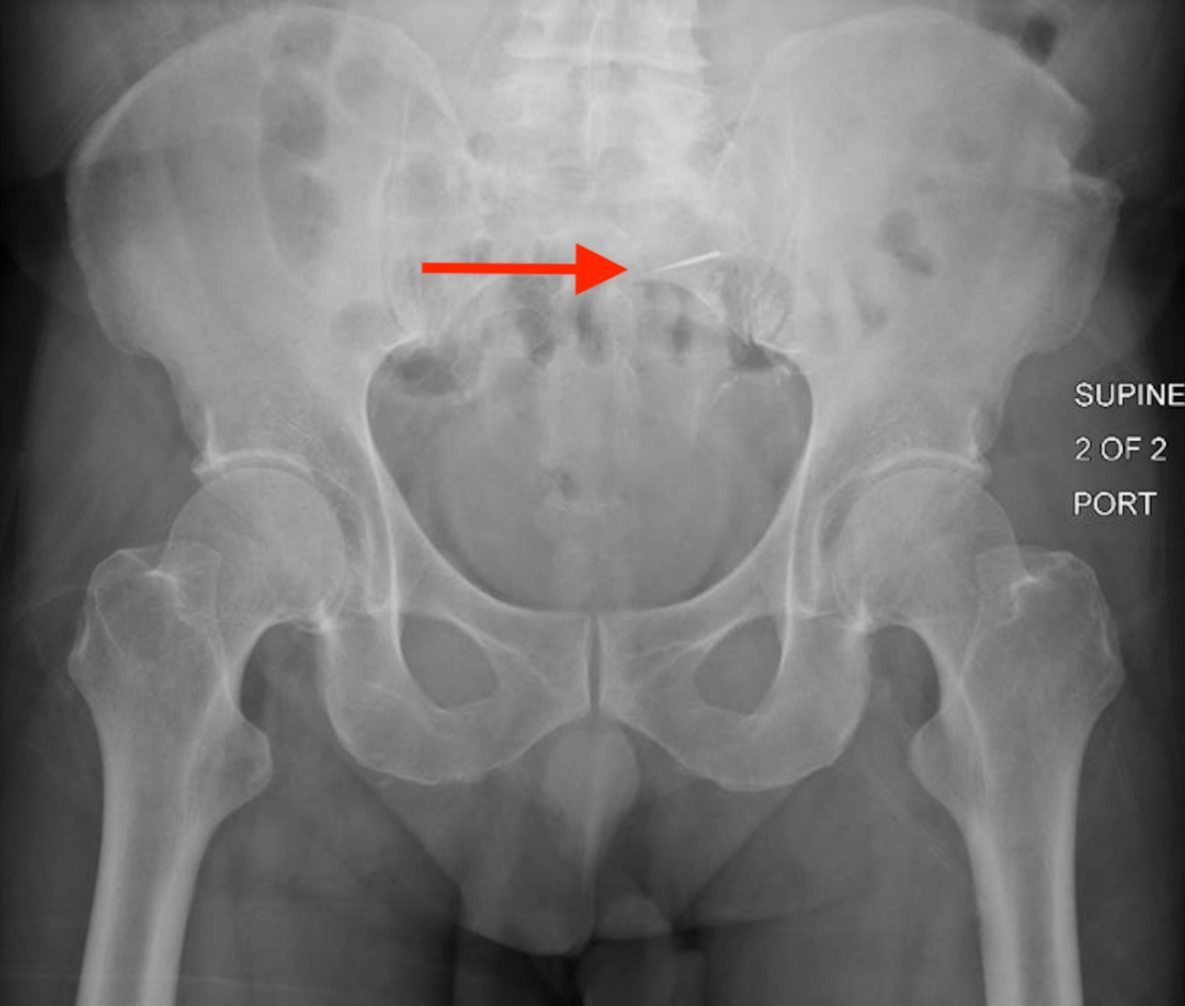
Repeat abdominal X-ray revealing a foreign body (red arrow) in the left upper pelvis.

**Figure 3:**
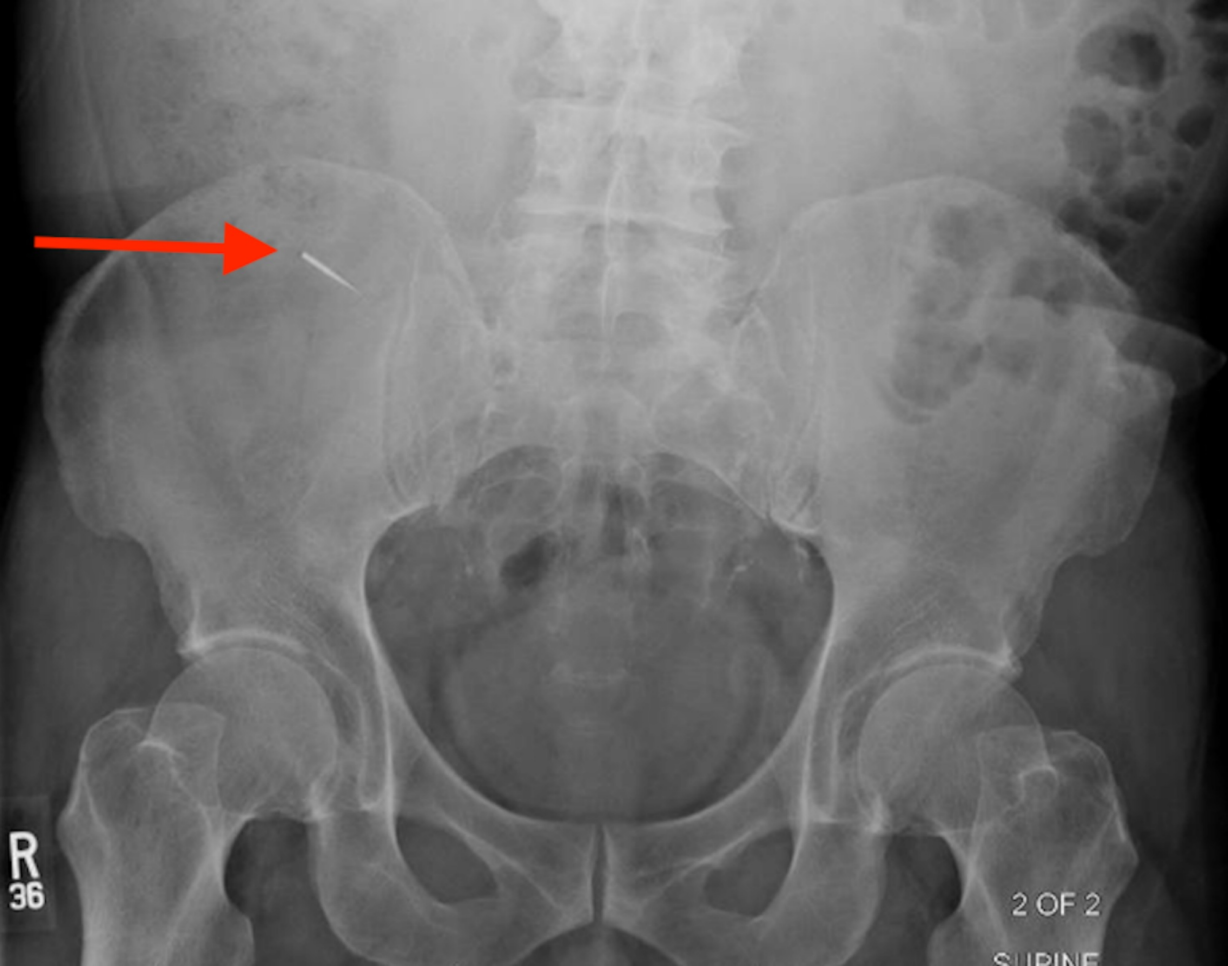
Final serial abdominal X-ray revealing a radiopaque foreign object (red arrow) in the right lower abdominal quadrant, presumed to be within the cecum.

The patient underwent an exploratory upper endoscopy with small bowel enteroscopy to attempt object retrieval, but the foreign object was not identified. Repeat abdominal X-ray after the procedure showed the drill bit to be in the left upper pelvis (Figure 2). Serial abdominal X-rays showed progression of the object along the GI tract. The final repeat image showed no further progression and the foreign body was located in the right lower abdominal quadrant, presumed to be within the cecum (Figure 3).

**Figure 4:**
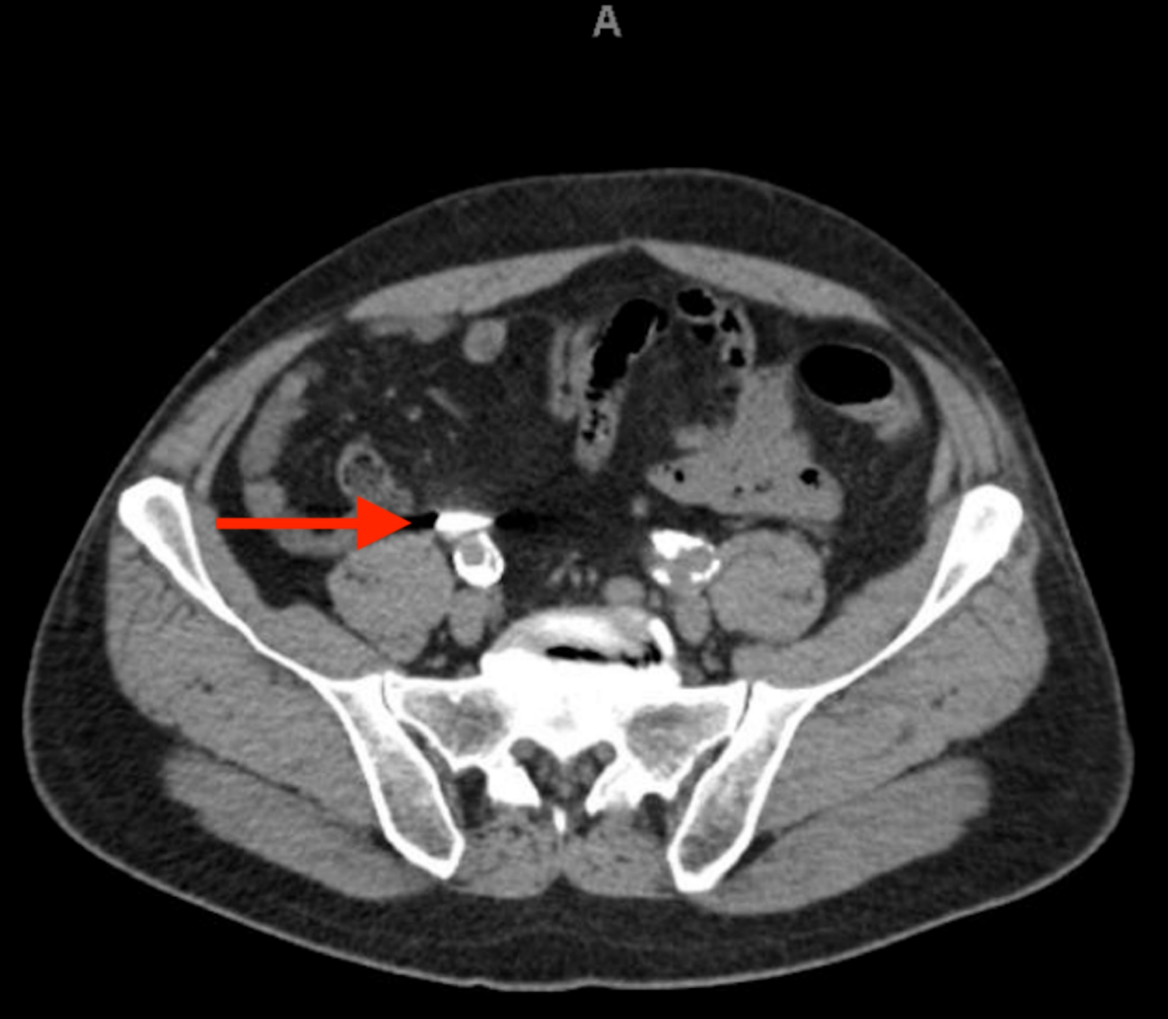
CT scan of the abdomen and pelvis with contrast revealing an intra-appendiceal dense foreign body (red arrow) with a distended and air-filled appendix.

An abdominal CT scan with contrast was obtained for better visualization, revealing an intra-appendiceal foreign body with associated appendiceal distention and air retention, consistent with appendicitis (Figure 4). The patient subsequently underwent laparoscopic appendectomy. Post-operatively, he was hemodynamically stable and was discharged on the same day without complication.

## Discussion

Appendicitis is thought to be incited by luminal obstruction of the appendix and subsequent accumulation of goblet cell secretions. The increased intraluminal pressure within the appendix leads to appendicular wall ischemia and vulnerability to abscess, peritonitis, and perforation [1]. While fecaliths are commonly implicated as the causative agents, this case highlights the importance of considering other etiologies of obstruction, including foreign bodies. Most foreign bodies will move through the GI tract without incident, however, objects may end up in the appendix due to gravitational dependence. Once foreign bodies are in the appendix, they are difficult to expel spontaneously. While foreign body-associated appendicitis is often asymptomatic, some patients may present with nonspecific symptoms, especially abdominal pain. The onset of clinical presentation of foreign body-associated appendicitis varies immensely, ranging from hours to years [4].

Initial management of foreign body ingestion includes imaging and urgent upper endoscopy to localize and, if possible, retrieve the object. If the foreign body cannot be reached via upper endoscopy, then repeat abdominal plain radiographs are warranted to monitor its progression along the GI tract. Radiographic signs suggestive of appendicitis include gas in the appendix, air-fluid levels or distention of the terminal ileum, cecum, or ascending colon, loss of cecal shadow, and free intraperitoneal air or fluid [2]. Clinicians may also consider obtaining complete blood counts to identify evidence of leukocytosis in the setting of asymptomatic appendicitis. Recently, using ultrasound to confirm foreign body-associated appendicitis has been gaining support as ultrasound imaging has been shown to decrease the number of unnecessary admissions and appendectomies [3].

If radiographic or ultrasonographic imaging reveals lodging within the appendix, it is recommended to remove the appendix prophylactically. Despite many patients being asymptomatic once the object is in the appendix, an appendectomy is still the standard of management to avoid inflammatory changes and perforation. This is particularly true for foreign bodies with sharp, thin, and non-flexible edges as these objects have been associated with perforation in 70% of cases [8]. There is more controversy surrounding the management of blunt objects as they are more likely to remain dormant for longer periods of time [9,10]. While there is no universal agreement among clinicians regarding management of these cases, approximately 66% of these patients eventually become symptomatic [8]. Thus, lack of symptoms in the setting of an appendicular foreign body should not affect one’s decision to act prophylactically with laparoscopic appendectomy. Post-operative care should follow the same protocol as that of common acute appendicitis unless complications were noted.

## Conclusions

We present a rare case of asymptomatic appendicitis secondary to ingestion of a dental drill bit. Despite the uncommon nature of a foreign body lodging within the appendix, this case highlights the importance of considering foreign body ingestion as a cause of asymptomatic appendicitis. It is crucial to recognize foreign body ingestions lodging in the appendix because patients are at a higher risk of complications, including abscess, peritonitis, perforation and even death. With timely recognition, prophylactic appendectomy can ameliorate these risks and minimize associated morbidity with foreign body-associated appendicitis.
